# Associations between food groups and liver cancer: a systematic review and meta-analysis of observational studies

**DOI:** 10.1186/s12937-023-00858-5

**Published:** 2023-06-22

**Authors:** Ke Liu, Weiwei Chen, Yi Zhou, Liuhong Xu, Xiaohui Sun, Yingying Mao, Ding Ye

**Affiliations:** Department of Epidemiology, School of Public Health, Zhejiang Chinese Medical University, 548 Binwen Road, Hangzhou, 310053 Zhejiang China

**Keywords:** Liver cancer, Food groups, Meta-analysis, Dose-response

## Abstract

**Context:**

Diet is emerging as a modifiable component of lifestyle for influencing the incidence of liver cancer.

**Objective:**

To investigate and quantify the potential relationship between food groups and liver cancer.

**Data sources:**

PubMed and Web of Science were searched for eligible observational studies until 31st March, 2023.

**Data extraction:**

The meta-analysis was conducted by pooling relative risk (RR), odds ratio (OR) or hazards ratio (HR) with 95% confidence intervals (CIs). Potential sources of heterogeneity were detected by subgroup analysis. Sensitivity analysis and publication bias test were also carried out.

**Data analysis:**

Through stepwise screening, a total of 27 studies were included. The pooled estimates of liver cancer for whole grains and legumes intake were 0.66 (95% CI: 0.54–0.82; *I*^*2*^ = 25.3%) and 0.86 (95% CI: 0.75–0.99; *I*^*2*^ = 14.3%), respectively. However, there were null associations of nuts, poultry, egg and sweetened beverages consumption with liver cancer and the association between refined grains and liver cancer was inconclusive. In dose-response meta-analysis, the pooled estimates of liver cancer were 0.77 (95% CI: 0.65–0.91) for every 50 g/day increment in whole grains intake. Non-linear dose-response relationship (*P* = 0.031) was observed in the association between the intake of legumes and liver cancer, and the protective effect occurred with the dose ranging from 8 g/day to 40 g/day.

**Conclusions:**

This meta-analysis shows that whole grains and legumes were inversely associated with liver cancer, whereas intake of nuts, poultry, egg and sweetened beverages may not be associated with liver cancer. Further quantitative research needs to be undertaken within a range of populations to investigate the relationship between food groups and liver cancer.

**Systematic review registration:**

PROSPERO registration no. CRD42021246142

**Supplementary Information:**

The online version contains supplementary material available at 10.1186/s12937-023-00858-5.

## Background

Liver cancer is the sixth most frequently diagnosed cancer and the third leading cause of cancer-related death worldwide in 2020 [1]. The global incidence and mortality of liver cancer have been on the rise for the past 10 years, with more than 900,000 new cases diagnosed and more than 800,000 cancer deaths annually [1]. Also, liver cancer is the most common cancer in 11 geographically diverse countries that located in Eastern Asia, South- Eastern Asia, and Northern and Western Africa [1]. Considering an overall increasing burden of liver cancer, thus, it is important to identify risk factors of liver cancer to provide ideas for its prevention. The well-established risk factors for liver cancer include chronic viral hepatitis, metabolic liver disease, alcohol drinking, smoking, obesity, and exposure to carcinogenic substances such as aflatoxins [2]. Increasing evidence suggested that diet as a modifiable component of lifestyle is suspected to influence the incidence of liver cancer [3].

Eating patterns assessed by hypothesis-driven approaches such as the Mediterranean diet score and the healthy eating index were reported to be associated with the risk of liver cancer [4–6]. However, concentrating on specific food groups may help to understand the role of dietary factors play in the risk of developing liver cancer, which could be more easily communicated to the public and could form the basis for dietary recommendations for the prevention of liver cancer. The World Cancer Research Fund (WCRF) and the American Institute for Cancer Research (AICR) have recently released reports on the prevention of liver cancer through diet and physical activity [7]. There is limited but suggestive evidence that consuming fish, coffee, and dairy products may decrease the risk of liver cancer. The WCRF and AICR reports similarly suggested maintaining a healthy diet rich in fruits, vegetables, nuts and whole grains may reduce the risk of liver cancer. Despite the availability of these reports, up-to-date evidence about the association of liver cancer with consumption of grains, legumes, poultry, nuts, egg and sugar-sweetened beverages (SSBs) has not been synthesized. In addition, the results from different studies on the association of specific food groups and liver cancer are inconsistent [8]. Therefore, a thorough investigation regarding the impact of specific food groups on liver cancer is warranted to inform future dietary guidelines.

In the present study, we conducted a systematic review and meta-analysis of the association of specific food groups including grains (whole grains and refined grains), legumes, nuts, poultry, eggs and sweetened beverages with liver cancer to provide a better dietary instruction for the lay public.

## Materials and methods

The current meta-analysis was conducted according to the standards and recommendations set by the preferred reporting items for systematic reviews and meta-analyses (PRISMA) [9], and was registered in the international prospective register of systematic reviews (PROSPERO: CRD42021246142). The Preferred Reporting Items for Systematic reviews and Meta-Analysis (PRISMA) checklist for reporting the meta-analysis is shown in Supplementary Table S1.

### Search strategy and selection criteria

PubMed and Web of Science databases were systematically searched from the inception to 31st, March 2023. The search terms listed in supplementary table S2 were employed to retrieve the relevant articles. In addition, references of related studies were also checked to identify additional publications of interest.

Studies meeting the following criteria were included: (1) study type was an observational study; (2) information about the association for at least one of the following 6 food groups: grains (whole grains and refined grains), legumes, nuts, poultry, egg and sugar sweetened beverages on liver cancer; (3) directly reported odds ratio (OR) or hazards ratio (HR) or relative risk (RR) with 95% confidence interval (CI), or indirectly provided relevant data for calculation; (4) if study populations overlapped, the one with larger sample size was included. The exclusion criteria were as follows: (1) food groups included in published meta-analyses related to liver cancer in recent three years (including fruits, vegetables, dairy products, total meat, red meat, white meat, processed meat and fish) [10–12]; (2) animal study, review, meta-analysis, letter or comment; (3) no access to full text; (4) duplicate studies retrieved from various databases. Two reviewers (Liu K and Chen W) independently performed study review and inclusion, and discrepancies were solved by a third reviewer (Ye D).

### Data extraction and quality assessment

Data was extracted cross-checked by two researchers (Liu K and Xu L) independently from eligible studies. The extracted information included name of first author, publication year, data collection year, type of study design, country or region, sample size, dietary assessment method, type of food groups, type of liver cancer and variables adjusted or matched.

The Newcastle-Ottawa Scale (NOS) was used to evaluate the quality of included studies with scores ranging from 0 to 9 points [13]. This scale evaluates studies on the following aspects: (I) selection of cases and controls (4 scores); (II) comparability of cases and controls (2 scores); (III) ascertainment of exposure and non-response rate (3 scores). Studies with a quality score of no less than 7 points were considered as high quality. Two reviewers (Liu K and Zhou Y) assessed the study quality, and discrepancies were resolved by consensus and discussion.

### Statistical analysis

The multivariate-adjusted estimates were selected if they were reported in the original article; otherwise, the unadjusted estimates were calculated using the original data. In the categorical meta-analysis, ever intake of food groups was compared with non/occasional intake of food groups, which was defined by study-specific reference ranges. If the group of ever intake of food groups was set up into multiple categories, we combined the effect estimates into a single value in each study. For the food groups with less than 3 studies, no quantitative analysis was carried out.

The heterogeneity was evaluated by Q-statistic test and I-squared (*I*^*2*^) value [14, 15]. The random-effects model was used to pool the effect estimates, as the approach can be used whether or not there is heterogeneity [16]. Subgroup analyses were performed by year of publication, geographic location, quality score, sample size and study design. Furthermore, sensitivity analysis was used to check the stability of the pooled results by omitting one study at a time and combining the effect values of the remaining studies. Begg’s [17] test and Egger’s [18] test were used to evaluate publication bias.

For the significant association between specific food groups and liver cancer, we also pooled estimates comparing highest with lowest intake of food groups among the studies with equal or more than three different categories of intake of food groups. Furthermore, we conducted a two-stage dose-response meta-analysis [19]. Briefly, a restricted cubic splines model with four knots at fixed percentiles (5%, 35%, 65%, and 95% of exposure level) was used, which had negligible influence on the estimates [20]. If the original article provided exposure range but not the average or median, the midpoint of the upper limit and lower limit of the interval was regarded as the exposure level. When the highest category was open-ended, the width was assumed to be the same as that of the adjacent interval. In addition, we assigned zero as the lowest exposure dose when the lowest category was open-ended [20]. If the included studies used different units to assign the dose, we converted them into grams per day according to standard conversion from standard documents [21–23] (poultry: 1 serving = 100 g; nuts: 1 serving = 28 g; egg: 1 serving = 50 g).

All statistical analyses were performed using STATA version 11.0 and *P* < 0.05 was regarded as statistically significant.

## Results

**Fig. 1:**
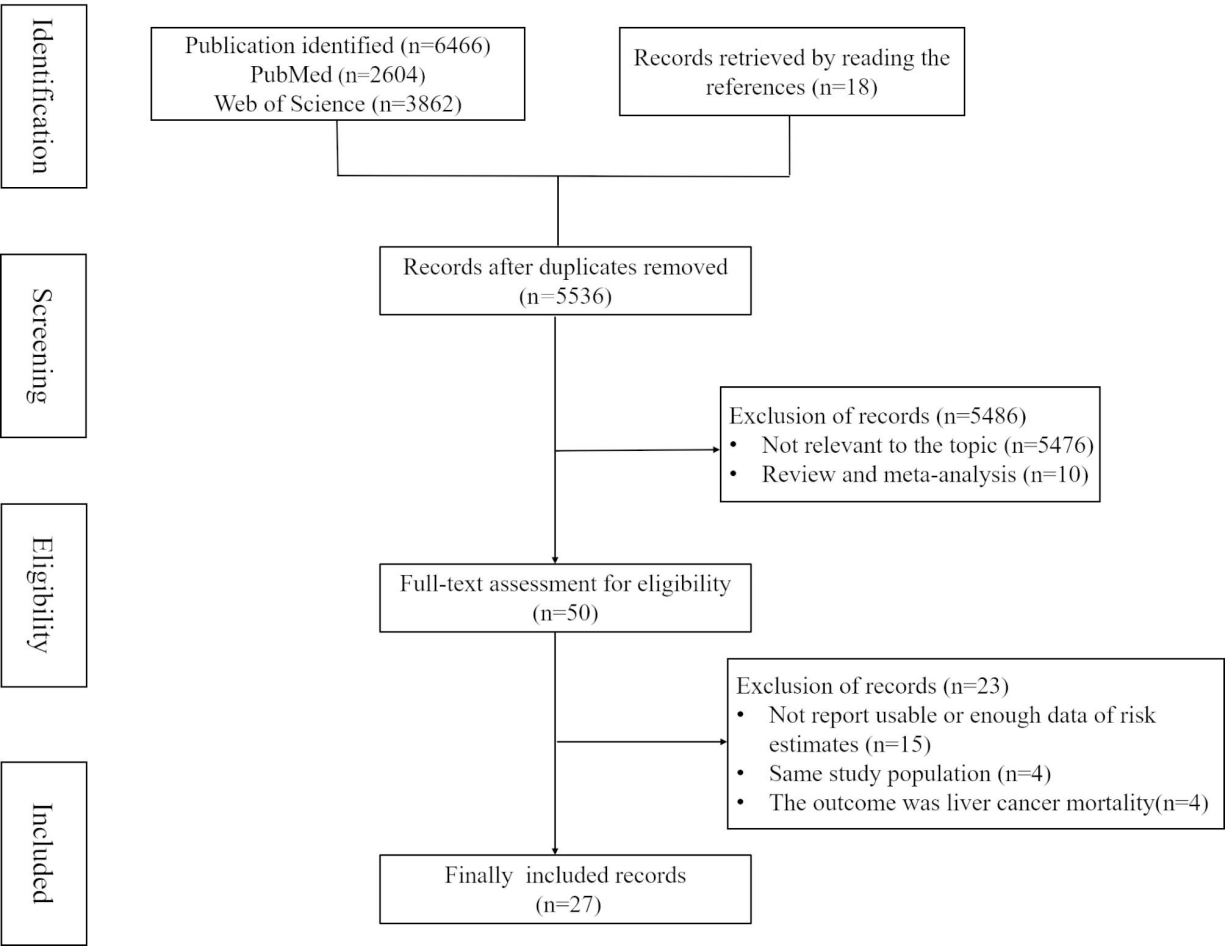
Flow-chart of study selection

The flowchart of the literature search is presented in Fig. 1. By searching databases of PubMed and Web of Science, and manually searching relevant references, a total of 6466 studies were searched, and 27 eligible studies were finally included based on selection criteria. Among these eligible articles, 12 were from Asia [24–35], 7 from Europe [36–42], 7 from America [43–49], and one from Africa [50]. There were 15 articles with prospective study [24, 29, 30, 32, 35, 39, 40, 42–49], and 12 articles with retrospective study [25–28, 31, 33, 34, 36–38, 41, 50]. The median quality score of all included articles was 7, which resulted from 16 articles with a score of 7 or more and 11 articles with a score of less than 7. Detailed characteristics of the included studies are shown in Table 1.

**Table 1:** Characteristics of included studies

Author, year	Country	Study type	Research period	Case/participants or controls	Dietary assessment method	Exposure details	Type of liver cancer	Adjusted	NOS	
Lam KC et al. [30], 1982	China	Retrospective	1977–1980	107/107	Dietary habits questionnaire	Legumes, nuts	HCC	Age, sex	5	
La Vecchia C et al. [34], 1988	Italy	Retrospective	NA	151/1051	FFQ	Egg, whole grains	HCC	Age, sex	5	
Hirayama T [21], 1989	Japan	Prospective	1966–1982	788/122,261	NA	Legumes	PLC	NA	5	
Chen CJ et al. [31], 1991	China	Retrospective	1985–1987	200/200	SFFQ	Nuts	HCC	Age, sex, ethnic group and residential area	6	
Srivatanakul P et al. [31],1991	Thailand	Retrospective	1984–1991	65/65	SFFQ	Nuts	HCC	NA	4	
Fukuda K et al. [23], 1993	Japan	Retrospective	1986–1992	368/485	SFFQ	Egg	HCC	Age, sex	5	
Zhang JY et al. [24],1998	China	Retrospective	1994–1995	152/115	SFFQ	Nuts	HCC	Liver disease (individual and family), drinking of alcohol, cigarette smoking, dietary aflatoxin intake, contact a toxic substance, blood transfusion, psychological distress, and infection with HBV and HCV	6	
Yu SZ et al. [25], 2002	China	Retrospective	1995–1997	248/248	SFFQ	Nuts, egg, poultry	HCC	Age, sex and residence	8	
Sharp GB et al. [26], 2005	Japan	Prospective	1964–1988	102/237	FFQ	Legumes	HCC	Sex, city, liver irradiation level, attained age, year of death, HBV and HCV	6	
Talamini R et al. [34],2006	Italy	Retrospective	1999–2002	185/412	FFQ	Egg, refined grains	HCC	Gender, age, centre, education, place of birth, drinking habits, maximal lifetime alcohol intake, hepatitis viruses (HBsAg and anti-HCV), and total energy intake	7	
Kurahashi N et al. [32],2009	Japan	Prospective	1993–2005	101/19,998	FFQ	Legumes	HCC	Age, area, HCV, HBsAg, smoking status, alcohol consumption, and intake of coffee and vegetables, menopausal status in women	8	
Kanazir M et al. [35],2010	Serbia	Retrospective	2004–2007	45/90	FFQ	Legumes, poultry	HCC	Age, sex	5	
Soliman AS et al. [47],2010	Egypt	Retrospective	2007–2009	149/150	SFFQ	Nuts	HCC	Age, sex	7	
Daniel CR et al. [40],2011	America	Prospective	1995–2006	582/492,186	FFQ	Poultry	HCC	Red meat intake, age, sex, education, marital status, family history of cancer, race, BMI, smoking status, frequency of vigorous physical activity, MHT in women, and intake of alcohol, fruit, vegetables, and total energy; mutually adjusted for intake of fish or poultry	9	
Fedirko V et al. [36], 2013	EPIC	Prospective	1992–2010	191/4,772,006	FFQ	Poultry	HCC	Smoking status, sex-specific physical activity level, self-reported diabetes status, lifetime alcohol intake pattern, BMI, baseline intakes of coffee, alcohol, dietary fiber and hepatitis viruses	9	
Zhang W et al. [27],2013	China	Prospective	1997–2011	267/132,837	FFQ	Legumes	PLC	Age, sex, body mass index, total energy intake, family income level, education level, family history of liver cancer, history of diabetes, history of cholelithiasis or cholecystectomy, vitamin C and E and multivitamin supplement use, and mutual adjustment for these dietary patterns	9	
Stepien M et al. [37], 2016	Denmark, France, Greece, Germany, Italy, the Netherlands, Norway, Spain, Sweden, and the United Kingdom	Prospective	1992–2010	191/477,206	Country-specific dietary questionnaires	Sweetened beverages	HCC	Non-alcoholic energy intake and stratified by age (1-year intervals), sex, study centre, BMI, sex-specific physical activity, education level, alcohol at recruitment and alcohol intake pattern, smoking intensity, duration and history diabetes status	8	
Rizk M et al. [38],2018	France	Retrospective	2008–2012	181/401	Diet history questionnaire	Legumes, nuts, egg, whole grains, refined grains, sweetened beverages	HCC	Age, gender, center, total energy from nonalcoholic sources, cirrhosis diagnosis, child-pugh score, diabetes, etiology of cirrhosis, alcohol consumption and occupational physical activity	6	
Luo X et al. [44], 2019	America	Prospective	1980–2012	160/137,608	FFQ	Sweetened beverages	HCC	Age (in months), study period (two-year interval), gender (women, men), race (White, non-White), physical activity (3, 3-<27, >=27 METS-hours/week), smoking status (never, past, current), body mass index (kg/m2, continuous), aspirin use (yes, no), alcohol intake (g/day, continuous), and total calorie intake (kcal/day, tertiles)	8	
Ma Y et al. [41], 2019	America	Prospective	1976–2012	163/142,857	FFQ	Poultry	HCC	Age, sex, race, physical-activity level, body mass index, smoking, type 2 diabetes, regular aspirin use, alcohol intake and total calorie intake	8	
Sui J et al. [42], 2019	America	Prospective	1980–2012	162/140,275	FFQ	Nuts	HCC	Age, gender, race, physical activity, BMI, smoking status, aspirin use, type II diabetes, total alcohol intake, total coffee intake, total calorie intake and hepatitis viruses	9	
Yang W et al. [43], 2019	America	Prospective	1984–2012	141/125,455	FFQ	Whole grains	HCC	Age (in months), race (white vs. nonwhite), physical activity level (metabolic equivalent of task–hours per week; continuous variable), body mass index (calculated as weight in kilograms divided by height in meters squared, continuous variable), smoking (0, 1 to < 10, 10 pack-years), regular aspirin use (yes or no); alcohol intake (< 5, 5 to < 15, 15 g/d), and type 2 diabetes (yes or no)	8	
Shawon MA et al. [28], 2020	Bangladesh	Retrospective	2018–2019	80/101	FFQ	Egg	HCC	Age, sex, income, and sociodemographic status	4	
Abe SK et al. [29], 2021	Japan	Prospective	1995–2013	534/75,089	FFQ	Legumes	HCC	BMI, history of type 2 diabetes, smoking status, alcohol consumption, coffee intake, and menopausal status in women	8	
Liu X et al. [45], 2021	America	Prospective	1995–2011	940/485,717	FFQ	Whole grains	PLC	Age(continuous), level of education (‘<=11 years’, ‘high school’, ‘vocational technology school’, ‘some college’, ‘college/postgraduate’), race (‘non-Hispanic white’, ‘non-Hispanic black’, ‘Hispanic’, ‘Asian, Pacific Islander, or American Indian/Alaskan native’), BMI (‘<25’, ‘25–30’, ‘30+’ kg/m2), alcohol use (‘non-drinker’, ‘0.1–4.9’, ‘5–9.9’, ‘10+’,gram/day), tobacco smoking (‘never smoked’, ‘former smoker’, ‘current smoker’), physical activity (‘never’, ‘rarely’, ‘1–3 time per month’, ‘1–2 times per week’, ‘3–4 times per week’, ‘5 + times per week’), history of diabetes (‘no’, ‘yes’) and total energy intake (continuous)	8	
Jones GS et al. [46], 2022	America	Prospective	NIH-AARP study: 1995–2011; PLOS:1993–2017	839/485,717	Dietary habits questionnaire	Sweetened beverages	PLC	Age at baseline, sex, race/ethnicity, body mass index, smoking, alcohol use, study, total energy intake (kcal/day).	8	
Guo W et al. [39], 2022	United Kingdom	Prospective	2006–2021	669/372,492	FFQ	Poultry	PLC	Age, sex, race, education level, Townsend Deprivation Index (quartiles), drinking status, smoking status, exercise, BMI and diabetes.	8	

FFQ: Food Frequency Questionnaire; SFFQ: Self-administered Food Frequency Questionnaire; PLC: Primary Liver Cancer; HCC: Hepatocellular carcinoma; EPIC: European Prospective Investigation into Cancer and Nutrition; NA: Not Applicable

### Grains

**Fig. 2:**
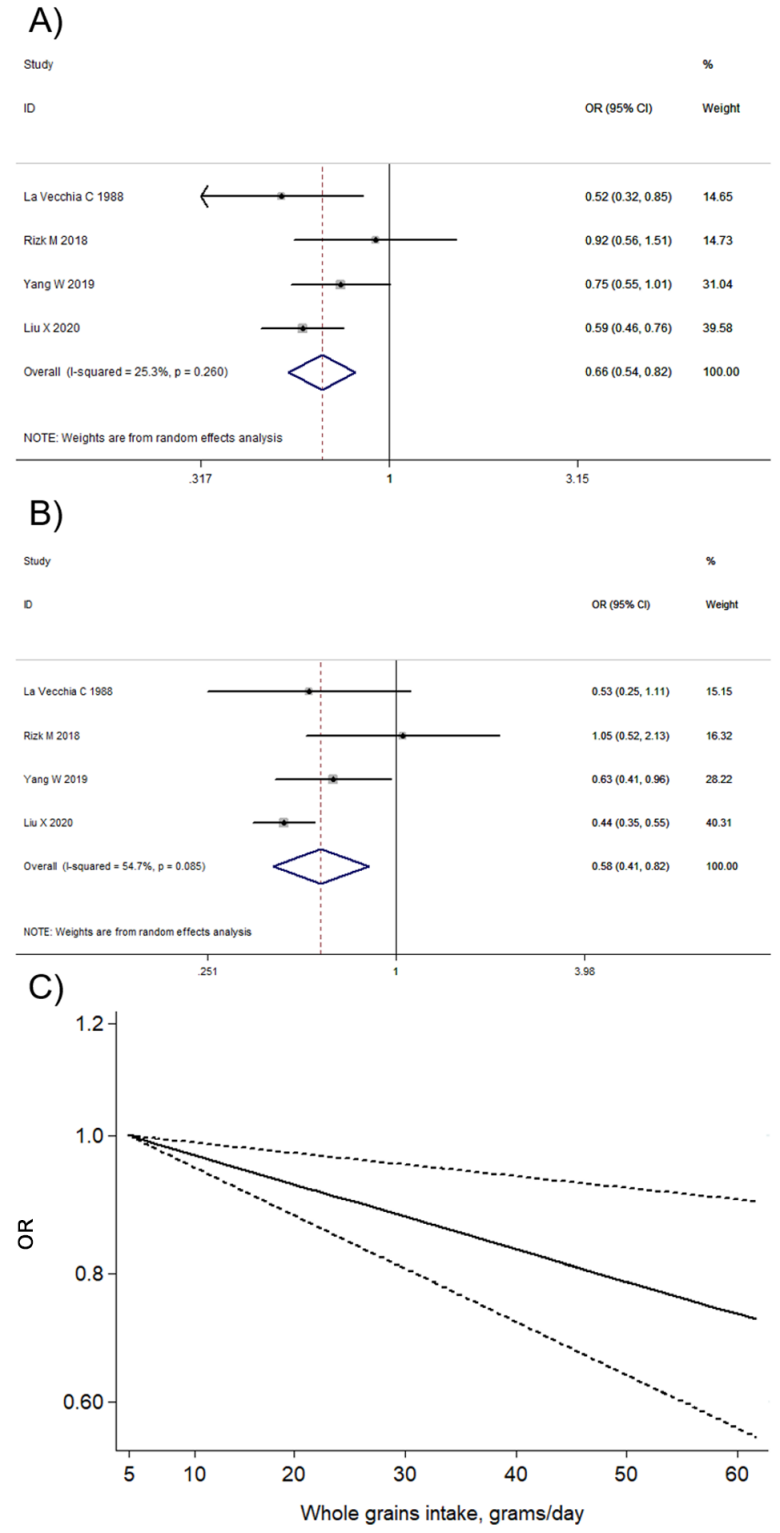
Association between whole grains intake and liver cancer comparing ever vs. non/occasional (**A**) and highest vs. lowest (**B**) whole grains intake; dose-response relationship between whole grains intake and liver cancer (**C**)

For whole grains, a total of 2 prospective studies [46, 48] and 2 retrospective studies [36, 41] with the sample size of 612,624 (1414 cases) were included to identify the association between whole grains intake and liver cancer. The summary estimates were 0.66 (95% CI: 0.54–0.82; *I*^*2*^ = 25.3%; Fig. 2A) for ever versus non/occasional whole grains intake.

Sensitivity analysis showed that exclusion of each study did not significantly change the overall results (Supplementary Fig. S1). No publication bias was observed by Begg’s test (*P* = 0.734) or Egger’s test (*P* = 0.721) (Supplementary Fig. S2). In the subgroup analysis, the significant associations were found among the studies in Asian and American populations, assessed with higher quality, larger sample size and prospective study design. Subgroup analysis of publication year showed similar results with main analysis (Supplementary Table S3).

The inverse association between whole grains intake and liver cancer remained significant for highest versus lowest whole grains intake (OR = 0.58, 95% CI: 0.41–0.82; *I*^*2*^ = 54.7%; Fig. 2B). A linear dose-response relation between whole grains intake and the liver cancer was detected, as depicted in Fig. 2C. The pooled estimate of liver cancer was 0.77 (95% CI: 0.65–0.91; *P* = 0.002; n = 2) per 50 g/day increase in whole grains intake. However, no evidence of a non-linear association was observed (*P* = 0.102 for non-linearity).

Few large prospective studies have reported the association between refined grains and liver cancer. Pasta as a refined grain was investigated in two case-control studies for association with liver cancer [37, 41]. A multicenter case-control study conducted in Italy in 1999–2002 [37], including 185 liver cancer cases, showed that intake of pasta average 5.25 servings unit/week was associated with higher liver cancer (OR = 2.47, 95% CI: 1.09–5.63). However, no association was observed between pasta intake and liver cancer in the Cirrhosis and Risk of Hepatocellular Carcinoma in the East (CiRCE) study [41]. Overall, further studies on the association of refined grains and its types with liver cancer are warranted.

### Legumes

**Fig. 3:**
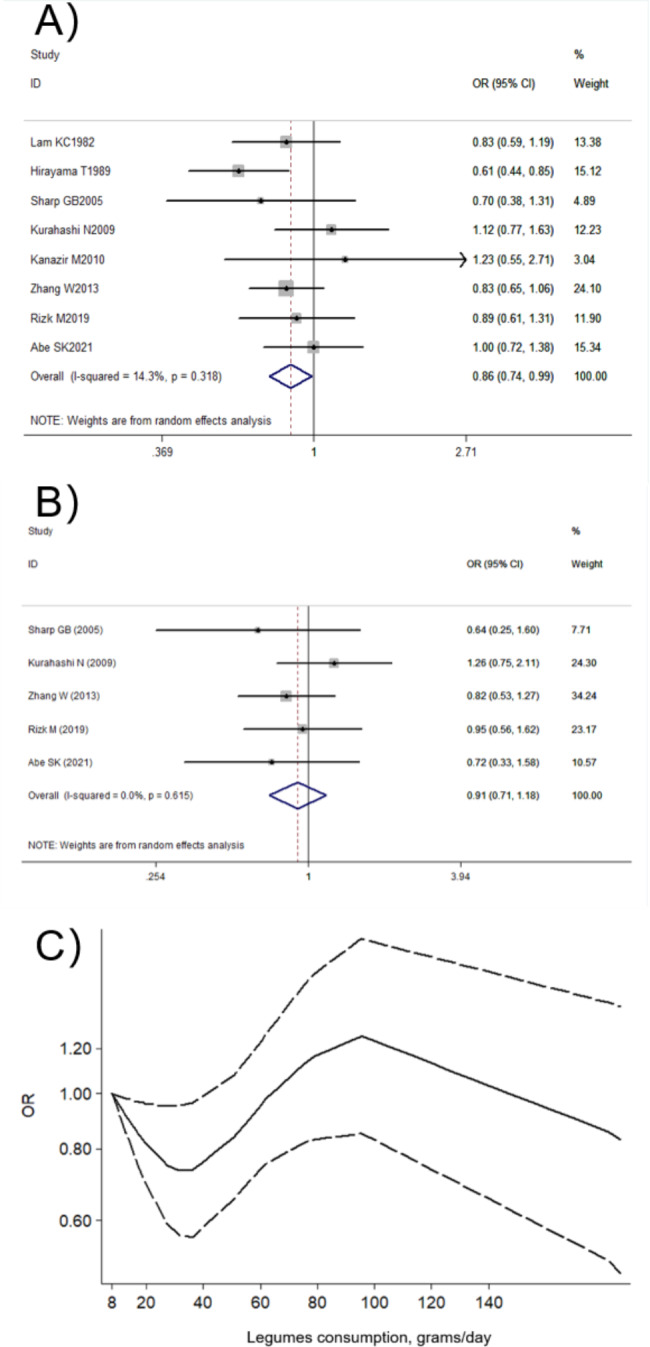
Association between legumes consumption and liver cancer comparing ever vs. non/occasional (**A**) and highest vs. lowest (**B**) legumes consumption; dose-response relationship between legumes consumption and liver cancer (**C**)

A total of 5 prospective studies [24, 29, 30, 32, 35] and 3 retrospective studies [33, 38, 41] among 351,931 individuals with 2125 cases were included to identify the association between legumes consumption and liver cancer. The summary estimates of liver cancer were 0.86 (95% CI: 0.75–0.99; *I*^*2*^ = 14.3%; Fig. 3A) for ever versus non/occasional legumes consumption. However, this association is not robust in sensitivity analysis (Supplementary Fig. S3). There was no sign of asymmetry with a *P* value of 0.711 by Begg’s test and 0.629 by Egger’s test (Supplementary Fig. S4). In the subgroup analysis, the significant inverse associations were found only among the Asian populations and lower quality studies (Supplementary Table S4).

The association between legumes consumption and liver cancer was insignificant when comparing highest versus lowest legumes consumption (OR = 0.91, 95% CI: 0.71–1.18; *I*^*2*^ = 0.0%; Fig. 3B). The nonlinear dose-response relationship curve (*P* = 0.031 for non-linearity; n = 4) showed that decreased liver cancer was observed when legumes intake ranged from 8 g/day to 40 g/day, and the most protective effect (OR = 0.76, 95% CI: 0.56–0.96) was observed at the dose of 36 g/day legumes consumption, as depicted in Fig. 3C.

### Nuts, poultry, egg and sweetened beverages

No significant associations of nuts, poultry, egg and sweetened beverages with liver cancer were observed. For poultry, there was a significant association found only in the American population. For egg, the significant associations were found among the studies with European population, higher quality and larger sample size (Supplementary Table S7). Subgroup analysis for nuts and sweetened beverages showed that the association remained insignificant when stratified by confounding factors, which are shown in Supplementary Table S5 and S8.

## Discussion

In this systematic review and meta-analysis, we systematically assessed the associations between six priori defined food groups and liver cancer. Our results showed that whole grains and legumes food were inversely related with liver cancer, of which the marginal association of legumes should be interpreted with caution. However, we have not found significant associations of nuts, poultry, egg and sweetened beverages intake with liver cancer, and the association between refined grains and liver cancer was inconclusive.

For whole grains, there was a negative association with liver cancer. The linear dose-response analysis showed that each additional daily 50 g whole grains intake was associated with a 23% decreased liver cancer. Even though the analysis was based on a small number of studies (4 studies in the pooled analysis and 2 studies in the dose-response analysis), subgroup analysis suggested that the protective effect of whole grains on liver cancer were consistently found in high-quality and large-scaled studies. Increased intake of whole grains and their component bran has been reported to have beneficial effects on diseases related to liver disease and liver cancer, including glycemia, insulin sensitivity, metabolic regulation, and reduced inflammation, etc [51–55]. Therefore, increasing intake of whole grain and bran may protect against liver cancer by mitigating the carcinogenic effect of hyperinsulinemia and inflammation. Also, experimental studies showed that whole grain may exert its potential antitumor (including cancers of colorectum and liver) activity through improvement of gut integrity and alteration of gut microbiota composition [56–58]. The biologic mechanisms for the inverse associations of whole grains with the liver disease remain to be fully elucidated.

Interestingly, we found that a reverse association of legumes and liver cancer with a low heterogeneity. Furthermore, the dose-response relationship meta-analysis suggested that there was a statistically significant association between legumes consumption and liver cancer at a certain dose range of 8-40 g/day. However, given the lack of robustness of the result and the fact that most studies focused on Asian populations, further evidence is needed in larger cross-population cohort studies in future. Regarding the potential mechanism, legumes contain a variety of phytochemicals, such as phytoestrogens, mostly isoflavone (genistein and daidzein) and lignans as well as saponins and phytosterols [59]. Anticancer effect of long-term genistein intake has been linked to suppressing hepatocellular carcinoma initiation and development through AMPK-mediated anti-inflammation and pro-apoptosis [60]. Besides genistein, legumes saponins may produce the anticancer effect through inhibition of hepatocellular carcinoma cells growth, direct cytotoxity, induction of apoptosis, antiestrogenic activity, etc [61–64]. These mechanisms might support the notion that legumes food intake was negatively associated with liver cancer incidence.

For nuts, most of tree nuts contain multiple hydrophilia compounds (quercetin, resveratrol, and ellagic acid) as well as lipophilic components (tocopherols, tocotrienols, omega-3, and omega-6 fatty acids), which have been shown to exert indirect anticancer activities via their anti-inflammatory and antioxidant actions [65]. A recent study showed that high consumption of nuts was significantly associated with decreased risk of overall cancer, especially apparent against cancers of the digestive system [66]. However, our study did not support the association between total nuts consumption and liver cancer. In terms of poultry intake, poultry as white meat is considered to provide suitable terrestrial sources of n-3 long chain polyunsaturated fatty acids (n-3 PUFA) [67], which has the properties of anti-inflammatory and anti-carcinogenic [68–70]. Although previous two prospective studies found the inverse association between poultry intake and liver cancer [43, 44], the possibility that residual confounders are responsible for the observed inverse association simply cannot be excluded because a high intake of poultry often clusters with a healthier overall eating pattern and lifestyle [71]. Additionally, we did not observe significant associations of egg and sweetened beverages with liver cancer based on a small number of studies. The varying results on these food groups can be attributed to the amount and type of food consumed, the different study sample sizes, demographics. Because of these inconsistencies further research will provide important evidence.

The present study also has several limitations. First, it is worth noting that residual components may influence the association of these food groups with liver cancer. For example, fermented beans than unfermented beans, sugar sweetened beverages than artificially sweetened beverages. Similarly, limited studies have provided information on the histopathological type of liver cancer, and therefore, we could not perform subgroup analyses or conduct the analysis separately according to these factors since preliminary studies reported limited or unclear results for food composition and liver cancer type. Secondly, meta-analysis across ethnic populations often leads to heterogeneity of findings because they have different dietary backgrounds, and statistically significant heterogeneity among studies was observed. However, the use of random-effects model was allowed to take the heterogeneity among studies into account. Thirdly, relatively few studies on some food groups are available and insufficient for further quantitative analysis, and there is no detailed data for dose-response analysis of the association between poultry consumption and liver cancer. Fourthly, the included studies are at risk of selection bias for cases and controls and the potential misclassification introduced by the lack of specificity in exposure definition. Moreover, insufficient adjustment for potential confounders (e.g., HBV/HCV status, total energy intake etc.) is also an important consideration on account of the nature of observational studies. Finally, the cutoff value of distinguishing between high and low consumption of food groups were diversiform in the included studies, which might contribute to the heterogeneity among the studies.

## Conclusion

In summary, our results demonstrated that whole grains, and legumes were inversely related with liver cancer. A null association was noted between nuts, poultry, egg and sweetened beverages consumption and liver cancer. Therefore, further well-designed cohort or clinical studies are needed to confirm the association.

## Electronic supplementary material

Below is the link to the electronic supplementary material.

Supplementary Material 1

## Data Availability

The datasets used and/or analyzed during the current study are publicly available and accessible.

## Abbreviations

BCAA: Branched-chain amino acid
CI: Confidence interval
HCC: Hepatocellular Carcinoma
HR: Hazards ratio
NOS: Newcastle-Ottawa Scale
OR: Odds ratio
RR: Relative risk
